# Impact of 12-month cryopreservation on endogenous DNA damage in whole blood and isolated mononuclear cells evaluated by the comet assay

**DOI:** 10.1038/s41598-020-79670-8

**Published:** 2021-01-11

**Authors:** Mirko Marino, Letizia Gigliotti, Peter Møller, Patrizia Riso, Marisa Porrini, Cristian Del Bo

**Affiliations:** 1 Division of Human Nutrition, Department of Food, Environmental and Nutritional Sciences (DeFENS), Università degli Studi di Milano, 20133 Milan, Italy; 2 Department of Public Health, Section of Environmental Health, University of Copenhagen, 1014 Copenhagen K, Denmark

**Keywords:** Biological techniques, Biomarkers

## Abstract

The comet assay is an electrophoretic technique used to assess DNA damage, as a marker of genotoxicity and oxidative stress, in tissues and biological samples including peripheral blood mononuclear cells (PBMCs) and whole blood (WB). Although numerous studies are performed on stored samples, the impact of cryopreservation on artifactual formation of DNA damage is not widely considered. The present study aims to evaluate the impact of storage at different time-points on the levels of strand breaks (SBs) and formamidopyrimidine DNA glycosylase (Fpg)-sensitive sites in isolated PBMCs and WB. Samples were collected, aliquoted and stored at − 80 °C. DNA damage was analyzed on fresh samples, and subsequently on frozen samples every 2 months up to a year. Results have shown no changes in DNA damage in samples of PBMCs and WB stored for up to 4 months, while a significant increase in SBs and Fpg-sensitive sites was documented starting from 6-month up to 12-month storage of both the samples. In addition, fresh and frozen WB showed higher basal levels of DNA damage compared to PBMCs. In conclusion, WB samples show high levels of DNA damage compared to PBMCs. One-year of storage increased the levels of SBs and Fpg-sensitive sites especially in the WB samples. Based on these findings, the use of short storage times and PBMCs should be preferred because of low background level of DNA damage in the comet assay.

## Introduction

The single-cell gel electrophoresis, best known as comet assay, is used to assess DNA damage at the level of individual cells^1,2^. It is versatile, quite simple, sensitive and non-invasive requiring only small aliquots of biological samples. In addition, it is also fast to give results, inexpensive and reproducible^2^. All these features allowed it to become a reference method and to be used in numerous in vitro and in vivo studies including biomonitoring studies^3,4^, clinical trials^5,6^ and dietary intervention studies^7,8^ in order to detect different types of damage such as single and double-strand DNA breaks, alkali-labile lesions, DNA–DNA/DNA–protein cross-links, and incomplete excision repair sites^9^. Additional steps in the protocol allow to obtain information about oxidatively generated DNA damage such as oxidized purines and pyrimidines by using specific endonucleases, or to evaluate DNA resistance to oxidative stress by using an oxidative insult (e.g. hydrogen peroxide)^10^. Considerable interest is well-deserved to all those DNA alterations since they underpin the mechanisms related to the main chronic-degenerative diseases, such as cancer, atherosclerosis, osteoarthritis and Alzheimer’s disease.

The comet assay is usually performed on isolated leukocytes or peripheral blood mononuclear cells (PBMCs) in human biomonitoring studies^11^. However, isolation of PBMCs is not always possible due to technical and practical reasons. For instance, a potential limitation may derive from studies involving numerous volunteers giving blood samples on one day and the consequent impossibility to isolate PBMCs. Isolation of leukocytes for the comet assay requires centrifugation steps and lysis of erythrocytes. A more attractive procedure is to simply embed whole blood (WB) in agarose and process it in the comet assay. The comet assay on WB requires only few microliters of blood; however, its applicability is under debate^12,13^.

Using fresh samples is the ideal procedure for the comet assay, because there are no concerns about artificial generation of DNA damage during the processing of samples. An alternative solution to the use of fresh samples consists in working with frozen material. There are advantages to use cryopreserved samples; they can be analyzed in whole batches, as opposed to blood samples that are procured over a period of time in a biomonitoring study and therefore entails some kind of storage before they are processed in the comet assay at even inconvenient times of the day. However, it is also useful in studies using repeated samplings from the same individuals such as intervention trials (i.e. sampling before and after a certain treatment). In these studies, it is variation over time that is the primary statistical outcome, and issues related to inter-assay variation are avoided by analyzing all frozen samples from each subject in the same comet assay experiment. Cryopreservation medium is widely used to preserve structurally intact cells for extended periods of time at very low temperatures. Storage may be applied both to PBMC and WB samples by keeping them at − 80 °C or under liquid nitrogen prior to identification of the best cryopreservation protocols to limit the effect of storage conditions on artifactual formation of damage. In this regard, there are different approaches to sample cryopreservation depending also on the biological matrix. For example, for large volumes of WB it has been suggested to dilute the sample with an equal volume of cell culture medium containing 20% dimethyl sulfoxide (DMSO, a cryoprotectant) or alternatively, to freeze directly small volumes of blood (max 250 µL) without cryopreservation solutions. These two approaches allow a rapid freezing process and reduce the risk of ice crystals formation and damages^3^. Regarding PBMCs, some researchers suggest suspending cells in a buffer or medium containing 10% DMSO^3^, while others report suspending cells in a storage medium containing RPMI-1640 medium, fetal bovine serum (FBS) and DMSO in the ratio 50:40:10 (v/v/v)^12^ or again using a medium containing 90% FBS and 10% DMSO^13^. In addition, for optimal cryoprotection of PBMCs, slow freezing is highly recommended by using commercial devices (e.g. Mr Frosty) that offer the ideal cooling rate close to minus 1 °C/min required for effective cryopreservation of cells. Also, the number of cells stored represents an important parameter to standardize; different studies recommend to freeze PBMCs at concentrations of ≤ 3 × 10^7^ cells/mL^3^.

However, the possibility to use fresh or cryopreserved WB and/or PBMCs is under debate due the lack of standardized e procedures that may represent an additional source of variability in the evaluation of DNA damage as already widely observed in several studies^14–18^.

In addition, there is disagreement among trials regarding the effect of storage on DNA damage^18–27^ as summarized in Table 1. Currently, the information available is quite limited and insufficient to exclude a possible impact of cryopreservation on the formation of artefacts that mask the real levels of damage^20,21,23,28^. These factors may represent a potential weakness especially when the comet assay is applied for evaluation of the impact of diets and/or dietary components on the protection against DNA damage as previously reported^21,24^. To partially overcome this problem, it has been recently published a consensus statement for the Minimum Information for Reporting Comet Assay (MIRCA) providing a list of recommendations to report in the papers for describing comet assay conditions^29^. Since cryopreservation and thawing procedures might increase DNA damage, it is highly recommended to include a description on the storage conditions as well as the freezing method and thawing procedure adopted.

**Table 1:** Summary of the effect of cryopreservation on DNA damage performed by comet assay in whole blood and different isolated human cells.

References	Sample	Cryopreservation method				Method of thawing	Result
		Temperature (°C)	Time	Volume of sample (µL)	Cryoprotectant		
Akor-Dewu et al.^18^	PBMCs	− 20	11 months	1000	Yes. Freezing medium (DMEM with 20% FBS, 10% DMSO)	Not reported	⬆ SBs in fresh compared to cryopreserved PBMCs
	PBMCs	− 80	11 months	1000	Yes. Freezing medium (DMEM with 20% FBS, 10% DMSO)	Not reported	⬆ SBs in fresh compared to cryopreserved PBMCs
	WB	− 20	11 months	200	No	Not reported	⬆ SBs in cryopreserved compared to fresh whole blood
Al-Salmani et al.^19^	WB	− 80	1 month	250	No	Thawed for up to 30 min at 4 °C	⬌ DNA damage
	WB	− 20	1 week	250	No	Thawed for up to 30 min at 4 °C	⬌ DNA damage
Del Bo' et al.^20^	PBMCs	− 80	12 months	1000	Yes. Freezing medium (50% FBS, 40% RPMI 1640 and 10% DMSO)	Gently thawed in a water bath at 37 °C and centrifuged to remove the medium	⬆ background DNA damage
							⬆ Fpg-sensitive sites
							⬇ H_2_O_2_ -induced DNA damage in cryopreserved compared to fresh PBMCs
Duthie et al.^21^	Lymphocytes	− 80	Up to 2 months	500 µL	Yes. Freezing medium (90% v/v heat-inactivated FBS and 10% v/v DMSO)	Rapidly thawed at 37 °C and immediately centrifuged to remove the freezing medium	⬌ DNA damage
Gajsky et al.^22^	WB	− 80	Up to 5 years	200–400	No	Quickly thawed in a 37 °C water bath	⬌ DNA damage
Ho et al.^23^	Lymphocytes	− 80	2–3 days and 4 weeks	500	Yes. Freezing medium (FBS and DMSO in a ratio of 9:1)	Quickly thawed in a 37 °C water bath	⬌ DNA damage
Koppen et al.^24^	WB	− 80	12 months	500	Yes. Freezing medium (70% RPMI 1640, 20% FBS and 10% DMSO)	Quickly thawed in a 37 °C water bath	⬌ DNA damage
	PBMCs	− 80	4–6 weeks	500	Yes. Freezing medium (70% RPMI 1640, 20% FBS and 10% DMSO)	Quickly thawed in a 37 °C water bath	⬌ DNA damage
Ladeira et al.^25^	WB	− 80	1, 4 and 12 weeks	200–250	No	Rapidly thawed at 37 °C	⬌ DNA damage
	PBMCs	− 80	1 and 4 weeks	200–250	Yes. Freezing medium (10% DMSO, 40% RPMI 1640 and 50% FBS)	Rapidly thawed at 37 °C	⬌ DNA damage
		− 80	12 weeks	200–250	Yes. Freezing medium (10% DMSO, 40% RPMI 1640 and 50% FBS)	Rapidly thawed at 37 °C	⬆ background DNA damage
							⬆ Fpg-sensitive sites
							⬆ H_2_O_2_ -induced DNA damage in cryopreserved compared to fresh PBMCs
Milic et al.^26^	WB	− 80	Up to 1 year	1000	No	Thawed for up to 5 min at 37 °C	⬌ DNA damage
Pu et al.^27^	White blood cells and Lymphocytes	− 20	1 and 7 days	Not reported	Yes. RPMI 1640 (10% FBS, 10% DMSO and 1 mM deferoxamine)	Not reported	⬌ DNA damage
		− 20	14 and 28 days	Not reported	Yes. RPMI 1640 (10% FBS, 10% DMSO and 1 mM deferoxamine)	Not reported	⬆ SBs
							⬆ DNA base oxidation in cryopreserved compared to fresh lymphocytes
		− 80	Up to 28 days	Not reported	Yes. RPMI 1640 (10% FBS, 10% DMSO and 1 mM deferoxamine)	Not reported	⬌ DNA damage
	WB	− 20	1 and 7 days	Not reported	Yes. RPMI 1640 (10% FBS, 10% DMSO and 1 mM deferoxamine)	Not reported	⬌ DNA damage
		− 20	14 and 28 days	Not reported	Yes. RPMI 1640 (10% FBS, 10% DMSO and 1 mM deferoxamine)	Not reported	⬆ SBs
							⬆ DNA base oxidation in cryopreserved compared to fresh whole blood
		− 80	Up to 28 days	Not reported	Yes. RPMI 1640 (10% FBS, 10% DMSO and 1 mM deferoxamine)	Not reported	⬌ DNA damage

*DMEM* Dulbecco's Modified Eagle Medium, *DMSO* dimethyl sulfoxide, *FBS* fetal bovine serum, *Fpg* formamidopyrimidine DNA-glycosylase, *PBMCs* peripheral blood mononuclear cells, *SBs* strand breaks, *WB* whole blood.

⬆, statistically significant high levels; ⬌, no statistically significant effect; ⬇, statistically significant low levels.

Within the cost action CA 15132 “The comet assay as a human biomonitoring tool (hCOMET)”, aiming to improve the analysis and reporting of results on DNA damage^30^, we decided to verify the impact of cryopreservation on the levels of DNA damage evaluated in WB and PBMC samples stored for 1 year and analysed every 2 months.

## Results

**Figure 1:**
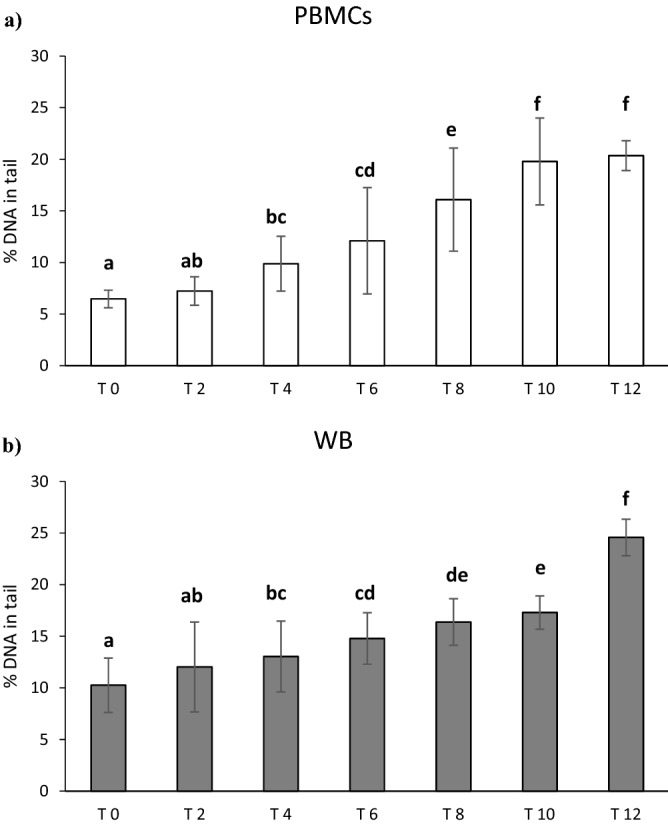
Effect of long-term cryopreservation on DNA strand breaks in PBMCs (**a**) and WB (**b**). T0 refers to fresh samples that have been processed in the comet assay without cryopreservation. T2–T12 are samples that have been cryopreserved between 2 and 12 months. Data are reported as mean ± standard deviation (SD); *PBMCs* peripheral blood mononuclear cells, *WB* whole blood. ^a,b,c,d,e,f^Data with different letters are significantly different (*p* < 0.05).

The impact of storage on DNA strand breaks (SBs) in PBMCs and WB is reported in Fig. 1a,b. ANOVA revealed that the time of cryopreservation increased the levels of background SBs both in PBMCs (*p* < 0.0001) and WB (*p* < 0.0001). In particular, the levels of background SBs in PBMCs (Fig. 1a) increased significantly from the 4th month up to 12th month of storage. The increase in DNA damage was continuous along time compared to baseline (fresh sample) and among each time point. While, no significant difference was observed from 6 to 8th month, and between 10 and 12th month. Similar to PBMCs, the levels of background SBs in WB (Fig. 1b) increased significantly from the 4th month up to 12th month of storage. The damage was always significantly different compared to baseline (fresh sample) and between each time point. At the end of the storage period, the increase in DNA damage was approximately + 140% compared to time zero.

**Figure 2:**
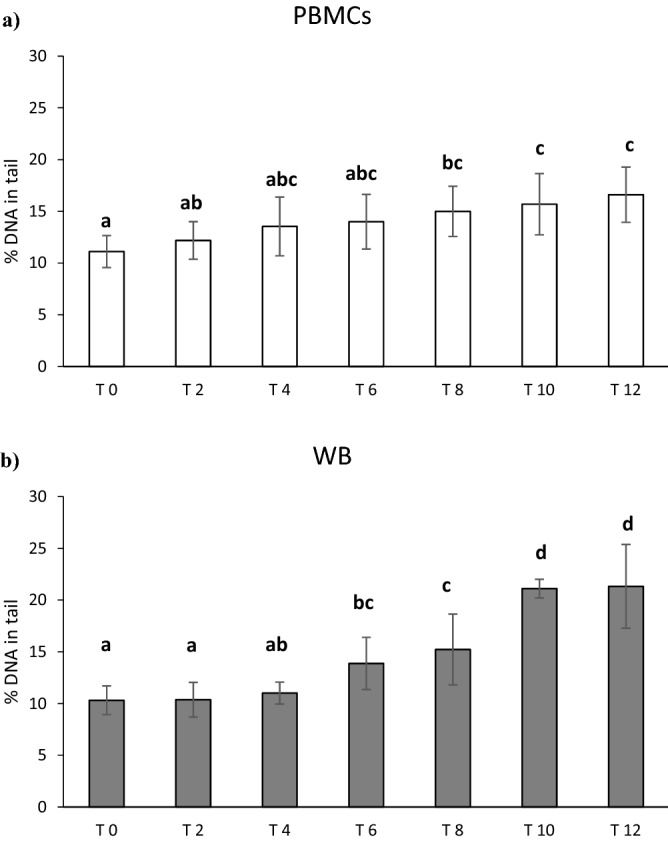
Effect of long-term cryopreservation on Fpg-sensitive sites in PBMCs (**a**) and WB (**b**). T0 refers to fresh samples that have been processed in the comet assay without cryopreservation. T2-T12 are samples that have been cryopreserved between 2 and 12 months. Data are reported as mean ± standard deviation (SD); *PBMCs* peripheral blood mononuclear cells, *WB* whole blood. ^a,b,c,d^Data with different letters are significantly different (*p* < 0.05).

Figure 2a,b reports the results of the impact of storage on Fpg-sensitive sites in PBMCs and WB. ANOVA showed a significant effect of time that increased the levels of damage both in PBMCs (*p* = 0.03) and WB (*p* < 0.0001).

In particular, in PBMCs (Fig. 2a) a significant increase was observed from the 8th month of cryopreservation (+ 34.9%; *p* = 0.020) up to 12th month (+ 49.5%; *p* = 0.002) compared to baseline, while no difference between 8 and 12th month was documented. Regarding WB (Fig. 2b), 4-month cryopreservation was enough to increase the levels of DNA damage compared to fresh samples (+ 34.5%; *p* = 0.04). A further significant increase in DNA damage was observed after 8 months compared to fresh sample (+ 105%; *p* < 0.0001) and 2th, 4th and 6th month (+ 91.7%, *p* < 0.0001; + 52.2%, *p* < 0.0001; + 38.6%, *p* < 0.0001, respectively). Following 8 months, no further significant augmentation was documented.

**Figure 3:**
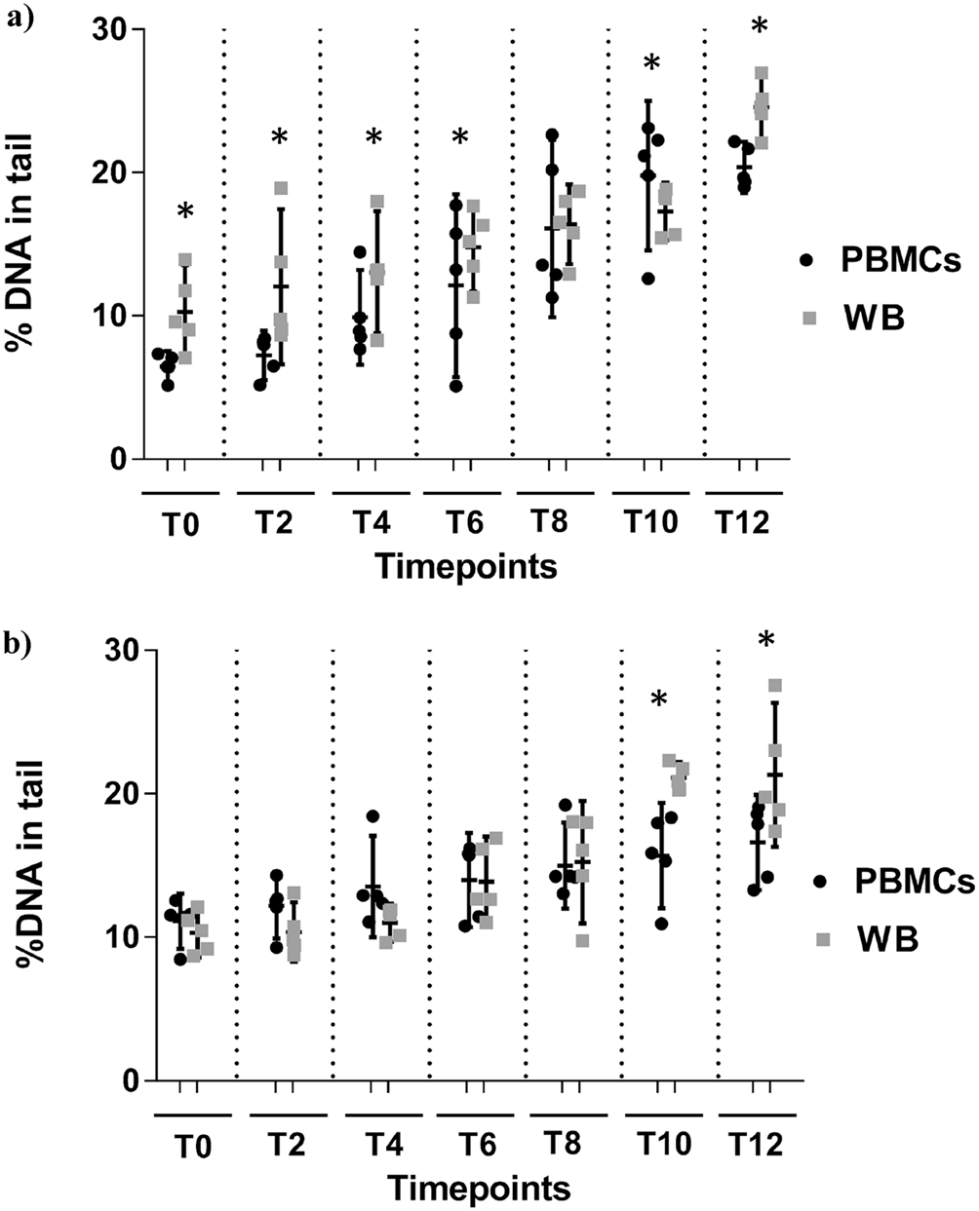
Comparison between PBMCs and WB for each of the replicates for (**a**) DNA SBs and (**b**) Fpg-sensitive sites. Results are displayed as percentage of DNA in tail along time (from time 0: fresh sample, till time 12 months of cryopreservation). *Significantly different (*p* < 0.05) within each time points between PBMCs and WB letters.

When comparing the levels of DNA damage between cells and blood, an effect of time (*p* < 0.0001) and treatment *x* time interaction (*p* = 0.001) was demonstrated for DNA SBs; in particular, PBMCs had lower levels of SBs with respect to WB both at baseline and within each time-points (Fig. 3a). Similarly, an effect of time (*p* < 0.0001) and treatment *x* time interaction (*p* = 0.004) was also documented for Fpg-sensitive sites. In this case, the levels of DNA damage were comparable between PBMCs and WB until 8-month storage and then resulted higher and significantly different at time 10 and 12 months in WB compared to PBMCs (Fig. 3b).

## Discussion

The evaluation of DNA damage by the comet assay is often performed on cryopreserved white blood cells such as PBMCs or sometimes subsets such as lymphocytes. However, there is evidence that the time of storage and the type of cryo-preservative might be important sources of variability in DNA stability due to artifactual damage as reported by Azqueta et al.^3^. In the current study, we have documented that the storage of PBMCs up to 6 months did not affect DNA stability, while extended cryopreservation has shown to increase the damage in line with our previous findings^20^. The results on short-term storage agree with those reported by Duthie et al.^21^ showing that lymphocytes can be successfully stored at − 80 °C up to 2 months without affecting DNA damage. Similar results were also reported by Ho et al.^23^, who found comparable levels of SBs and Fpg-sensitive sites between fresh and cryopreserved lymphocytes stored for 2–3 days and 4 weeks. Pu et al.^27^ found no significant changes in either direct DNA strand breakage or Fpg-sensitive sites in white blood cells and lymphocytes stored at − 20 °C for 1 and 7 days compared to fresh samples, while a significant increase was found for samples stored up to 14 and 28 days. This increase was mainly attributed to the temperature used; in fact, no changes in direct and oxidatively damaged DNA were observed when cells were stored at − 80 °C for up to 28 days. Conversely, Ladeira et al.^25^, showed a significant increase in DNA SBs following 1 and 4 weeks, but not 12 weeks, of PBMCs storage at − 80 °C, while no effect was observed on Fpg-sensitive sites. On the other hand, Akor-Dewu et al.^18^, reported high levels of SBs in fresh compared to cryopreserved PBMCs at − 80 °C and those stored at − 20 °C. Regarding the effect of long-term cryopreservation on the levels of DNA damage, Jackson et al.^31^ documented the feasibility to store, over a period of 1 year, both tissues and cells (A549 lung epithelial cells) reporting low variability (2–12% day-to day variability) and low levels of DNA strand break levels in the samples analysed. The discrepancies between studies could be attributed to the different type of cells used, time and temperature of storage, cryopreservation media, method of thawing washing and the centrifugation process. In this regard, the thawing process should be fast in cryoprotected cells to minimize DMSO cytotoxicity, whereas in non-cryoprotected samples slow thawing at 4 °C is advisable^14^ to minimize DNA damage arising from cell metabolic reactions. In addition, the temperature and speed of wash medium may affect the viability of the cells. Ramachandran et al.^32^ recommend to incubate frozen PBMCs at 37 °C from 10 to 30 min before their use. In addition, it is suggested to wash cells twice by slowly adding a pre-warmed medium (rate of 1 mL/ 5 s; 37 °C). However, other studies reported no washing process. For example, Moller et al.^33^ prepared cells (mouse lymphoma cells) in such a way that a vial could be collected from the freezer and directly embedded in agarose without centrifugation and resuspension of cells in fresh medium. In our experimental conditions, PBMCs were thawed at 37 °C and immediately processed in order to remove DMSO; however, no pre-warmed medium was used (medium at room temperature; 22 °C). Thus, we cannot exclude that the augmented levels in DNA damage observed may be related, at least in part, to the thawing and washing processes and/or the lack of pre-warmed medium.

Regarding the use of WB in the comet assay, several studies discussed its applicability, as a valuable alternative to PBMCs, in human biomonitoring. In fact, WB is a complex matrix constituted by different cells and materials such as erythrocytes, mononuclear as well as polymorphonuclear cells, platelets and plasma, and contains numerous components with potential antioxidant (i.e. albumin, bilirubin, uric acid), but also pro-oxidant activity (i.e. heme iron) making its use debated^22,24^. In addition, cryopreservation may represent an additional critical step for WB since the presence of water in plasma may determine the formation of intracellular ice crystals leading to freezing damage and the release of iron from erythrocytes that may cause the activation of oxidative processes. In this regard, several studies have been performed to identify the best approach of blood cryopreservation. Storage of large volumes (~ 5 mL) at − 80 °C, without cryo-preservative, have shown to increase the levels of damage, while the presence of DMSO has reported to minimize the detrimental effects of the freezing process^19,24,34^. Differently, the use of small volumes of WB seems to be more feasible also without adding a cryo-preservative. For example, Al-Salmani et al.^19^ have shown low levels of SBs and Fpg-sensitive sites in WB samples aliquoted in 250 µL and directly frozen at − 80 °C for 1 month. However, these authors reported problems in detecting DNA SBs after treatment with a high concentration of hydrogen peroxide (H_2_O_2_; 1 mM) probably due the breakdown of H_2_O_2_ by catalase. Similar findings were reported by Ladeira and coworkers^25^ showing no difference in DNA damage following 1, 4 and also 12 weeks of storage at − 80 °C of 200–250 µL WB samples. Gajsky et al.^22^, reported the possibility to store WB samples (200–400 μL) at − 80 °C, without cryoprotectant, even up to 5 years. Milic et al.^26^, showed no difference in DNA damage between fresh and stored blood (− 80 °C up to 1 year) when using a larger volume (1 mL) and without cryo-protection. Similarly, Akor-Dewu et al.^18^, reported no effect on samples stored in small aliquots (250 μL), with and without cryo-preservative, but for a maximum of few weeks or months. Conversely, Pu et al.^27^, found a significant increase in both SBs and DNA base oxidation in WB samples (volume not reported) stored at − 20 °C, with preservation medium, for 14 or 28 days, while no effect was observed after 1 or 7 days at − 20 °C, or at − 80 °C for up to 28 days. In the present study, we have reported that 250 μL of WB can be successfully stored, without cryo-protection for 4 months, while medium-long term storage (6–12 onths) showed to increase SBs and Fpg-sensitive sites. This discrepancy between studies could be attributed to different factors such as the volume of blood stored, the presence/absence of an appropriate freezing medium, the time and the temperature of cryopreservation, but also the thawing process (Table 1). This latter, seems in fact to affect the DNA integrity not only in isolated cells but also in WB. However, this information is not always provided along studies^18,27^. Some studies thawed samples at 37 °C^22,24–26^ while others at 4 °C^19^. Since there is still a lack of consensus regarding the thawing process, we cannot exclude that our process could have affected the results obtained.

Few studies have compared the levels of damage between PBMCs and WB. Here, we showed high levels of SBs damage in WB compared to PBMCs. This difference was already documented at baseline and within the different times, while no difference in Fpg-sensitive sites was observed until 8-month storage. Our results are in accordance with some studies^35–36^, but in contrast with others reporting no difference in SBs^37–38^ and Fpg-sensitive sites^25^ between WB and PBMCs. For example, Akor-Dewu et al.^18^ showed similar levels of SBs in fresh and frozen/thawed blood samples, while higher and remarkable differences were reported only for leucocytes isolated from frozen blood probably attributed to the processes of cell isolation. Similar findings were also documented by other authors^24,38^ but on fresh isolated PBMCs compared to fresh WB samples hypothesizing a contribution of the isolation methods in the damage observed. Thus, it is important to optimize the comet assay protocol based also on different cell type used and possibly considering the specific damage assessed^39^.

It is important to emphasize that the comet assay detects the migration of DNA in agarose gels and the preferred descriptor (%DNA in tail) or any other comet assay descriptors are indirect measures of genotoxicity. In fact, the percentage of DNA in the tail describes how much of the DNA that has migrated from the head to the tail, which is caused by breaks in the DNA strand. Its migration, rather than the number of lesions, are detected by the comet assay. The present study and other similar studies on the stability of cryopreserved cell samples have the inherent problem that they cannot control for variation over time because this is typically done using cryopreserved samples. Alternatives, such as using a cell line or blood cells from inbred rodents, might be considered as sufficiently stable, although there is uncertainty pertaining to these specimens too (e.g. even immortalized cells change when they are cultured too long). Thus, the upward trend in the level of %DNA in our study can be due to both a drift in the method (e.g. medium or equipment cause the DNA to migrate longer in the gels) and accumulation of DNA damage during storage. Unfortunately, we do not have an objective measure of the DNA stability that can be used as reference in these experiments. In our study, differences in SBs and Fpg-sensitive sites in PBMCs are 13% and 4% tail DNA, respectively (T12 versus T0). In perspective, the interquartile range of reference values for SBs and Fpg-sensitive sites in white blood cells have been estimated to 14.2% and 7.0% tail DNA^40^. Thus, the effect of long-term cryopreservation in the present study is within the range of DNA damage levels in white blood cells that are reported by other laboratories. The net increase of 13% and 4% SBs and Fpg-sensitive sites in PBMCs corresponds to approximately 2000 and 600 extra lesions in human diploid DNA, using calibration curves from the European Comet Assay Validation Group ring-trials^16,41^. Although, e.g. baseline levels of 20% tail DNA is relatively high, it is well within the linear dynamic range in our laboratory as e.g. demonstrated in calibration samples used in ring-trials^16,41^.

## Conclusions

In summary, taken together our results documented that WB and isolated PBMCs can be safely cryopreserved for only several months at − 80 °C because of the DNA damage progresses after longer storage. In this regard, it is advisable the use of liquid nitrogen for long-storage of PBMCs since their cryopreservation at − 80 °C does not represent a standard procedure. In fact, we have reported that medium-long term storage (from 6 up to 12 months) increased SBs and Fpg-sensitive sites suggesting a potential detrimental impact of freezing and/or thawing processes on DNA damage. This effect was more pronounced in WB samples compared to PBMCs, suggesting the importance to standardize the protocol not only in terms of type of cells (e.g. WB or PBMCs, fresh or cryopreserved) but also operational conditions (e.g. sampling, isolation, freezing, thawing). Based on our findings, the use of PBMCs should be advisable and preferred compared to WB since they represent a more homogeneous cell population. However, since the results from different studies are equivocal, further investigations are encouraged in order to provide more evidence on the impact of storage times on DNA damage in both the biological samples (WB and PBMCs), and to define the best protocol for their cryopreservation and thawing.

## Methods

### Chemicals

All the chemicals and reagents used for the separation of the samples and the analysis of the comet assay were purchased from Merck (Darmstadt, Germany). GelBond films were obtained from VWR International S.r.l (Pennsylvania, USA) while the enzyme Fpg was a gift from Prof. A.R. Collins (University of Oslo, Norway).

### Samples preparation and experimental design

The blood sample used (about 20 mL, two tubes) derived from a previous clinical trial testing the effect of polyphenol-rich foods on different markers related to human health, including DNA damage and other oxidative stress markers (trial registered number: ISRCTN10214981). The trial was performed in accordance with the principles reported in the 2013 Declaration of Helsinki and approved by the Ethics Committee of the University of Milan. An informed consent was provided and signed by all participants. For the study, an anonymized sample was used to test its stability during storage.

**Figure 4:**
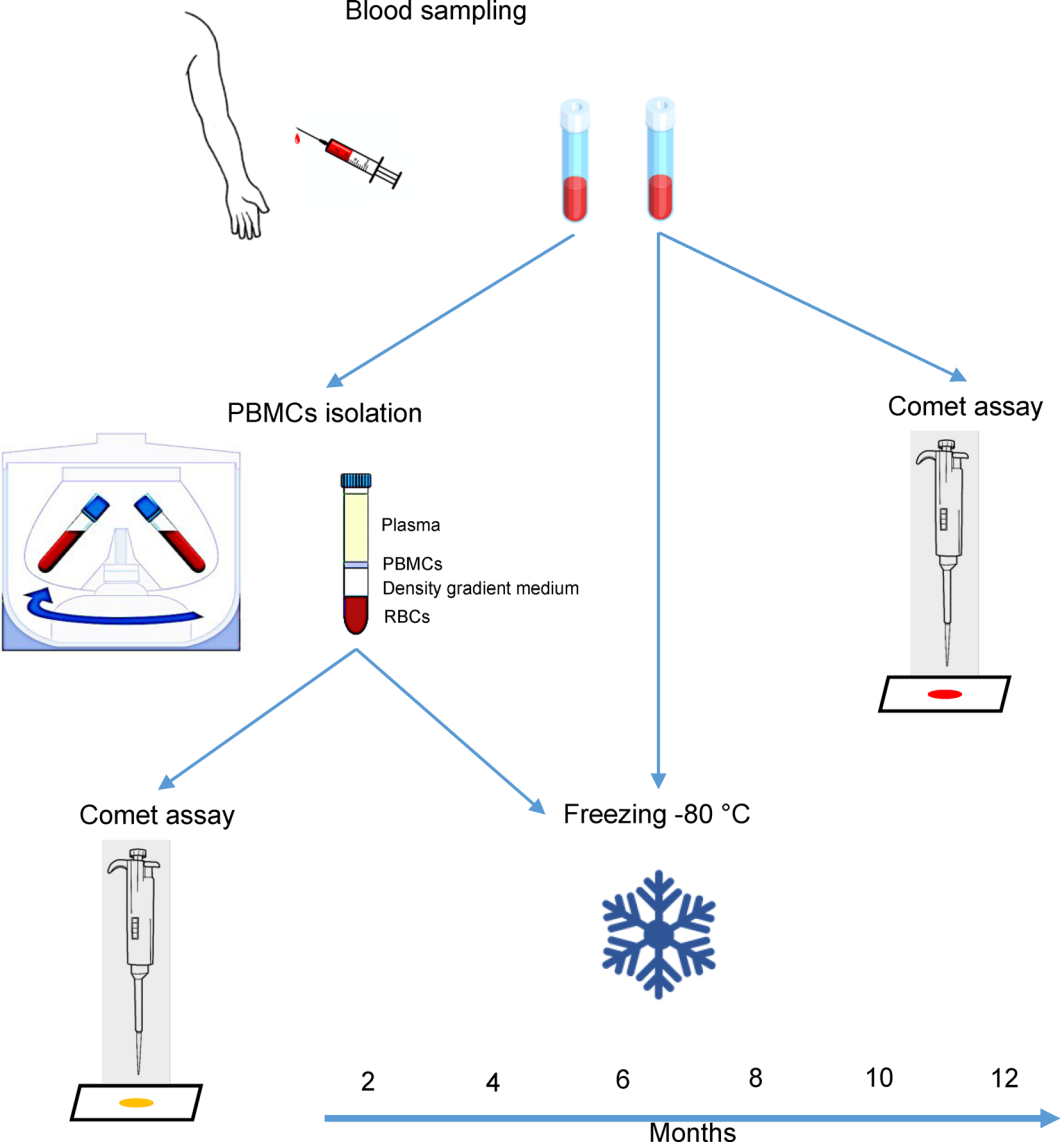
Experimental design. Blood is collected into two tubes containing EDTA as anticoagulant. One tube is used for the isolation of peripheral blood mononuclear cells (PBMCs) by a density gradient. Cells are washed, centrifuged and immediately used for the comet assay or aliquoted in an appropriate freezing medium and stored at − 80 °C. The second tube is used for whole blood (WB); a small aliquot (10 µL) is immediately used while the remaining samples are aliquoted in 250 µL, without cryo-preservative, and frozen. Every 2 months, comet assay is performed.

Blood was drawn into a Vacutainer containing ethylenediaminetetraacetic acid (EDTA) as anticoagulant and processed within 30 min after collection as summarized in Fig. 4. One tube was used for the aliquots of WB, while the other tube for the separation of PBMCs. Ten microliters of WB were immediately used for the comet assay, while the remaining part of the sample was aliquoted in 500 µL microcentrifuge tubes (containing each 250 µL of sample) without cryo-preservative^18,19^ and stored at − 80 °C up to 12 months. PBMCs were obtained after centrifugation by density gradient with Histopaque as previously reported^20^. An aliquot of PBMCs (1 mL) was used immediately for the comet assay while the rest of the samples were suspended in a freezing medium containing RPMI-1640, FBS and DMSO (50:40:10; v/v/v), aliquoted in 1.5 mL cryovials (containing 1 mL of cell suspension each). We have used this freezing medium for approximately 20 years in comet assay experiments on biomonitoring studies^42^, including studies on genotoxic effects of air pollution exposures^43^ and antioxidant interventions^44^. It was originally chosen as a cryopreservation medium because thawed cells had acceptable survival rate and responded well to stimulation with mitogens for cell proliferation. Samples were slowly frozen at − 80 °C by using a Mr. Frosty. Cells were maintained into a Mr. Frosty for 4 h before being transferred into a cryobox kept at − 80 °C up to 12 months. Every 2 months, an aliquot of WB and PBMCs was used for the analysis of DNA damage.

Blood was thawed on ice at room temperature (20–23 °C), while PBMCs at 37 °C for 10 min. After thawing, PBMCs were rapidly centrifuged at 5000×*g* for 10 s. in order to remove the freezing medium. Then, the pellet was washed with 500 µL of phosphate-buffered saline (PBS), centrifuged at 5000×*g* for 10 s. and resuspended in fresh PBS at the concentration of 1 × 10^6^ cells.

### Comet assay procedure and analysis

Ten microliters of fresh and frozen WB were diluted in 40 µL PBS and mixed with 160 µL of 1% LMP agarose (final concentration 0.7%) in PBS and two drops of 80 µL were applied onto large hydrophilic polyester films (GelBond), precoated with 1% NMP agarose, and covered with glass cover slips. Fifty microliters of fresh and cryopreserved isolated PBMCs were mixed with 160 µL of 1% LMP agarose (final concentration 0.7%) and 80 µL of samples were applied onto the GelBond film. For each sample of WB and PBMCs, 6 gels were prepared. Gels were covered with coverslips and stored for 5 min at 4 °C to solidify. After gel solidification, the slides were placed in lysis solution (2.5 M NaCl, 0.1 M Na_2_EDTA, 10 mM Tris and 1% Triton X-100, 1% DMSO, pH 10) for 1 h at 4 °C. Then, slides were washed 3 times in a buffer (40 mM HEPES, 0.1 M KCl, 0.5 mM EDTA, pH 8) before incubation with Fpg (100 ng/mL in buffer containing 0.2 mg/mL bovine serum albumin), or buffer only. During the incubation, the gels were covered with a coverslip and maintained in a humidified atmosphere, for 45 min at 37 °C. Successively, coverslips were removed and slides were transferred to an electrophoresis tank and covered with buffer (300 mM NaOH and 1 mM Na_2_EDTA, pH > 13) for 40 min at 4 °C before electrophoresis (20 min at 1.1 V/cm, 300 mA, 4 °C). Finally, the slides were neutralized in a buffer (0.4 M Tris–HCl, pH 7.5) for 15 min and dried in pure ethanol for 2 h. Slides were stained with ethidium bromide (2 μg/mL) and analyzed.

For each gel, 100 images were analyzed with an image analysis software (Cometa 1.5; Immagini e Computer, Bareggio, Milan, Italy). The percentage of DNA in the tail for each image was calculated. The level of Fpg-sensitive sites was calculated as the difference in % DNA in tail between the Fpg-treated cells and the control cells (the cells not treated with Fpg).

### Statistical analysis

Data were expressed as mean and standard deviation of % DNA in tail. Each experiment was performed in quintuplicate slides (each with 2 gels). Analysis of variance was used to evaluate the effects of sample (WB vs. PBMCs) as an independent factor and time of storage (0, 2, 4, 6, 8, 10 and 12 months) as dependent factors on background SBs and Fpg-sensitive sites. Differences between the mean values were evaluated by the least significant difference test with a level of significance of *p* ≤ 0.05. In the present study, *p* values indicate whether the variation over time (i.e. inter-assay variation) differs from the intra-assay variation (i.e. the variation between slides in the same experiment). All the analyses were performed using STATISTICA software (StatSoft Inc., Tulsa, OK, USA).
